# Dietary Complex and Slow Digestive Carbohydrates Prevent Fat Deposits During Catch-Up Growth in Rats

**DOI:** 10.3390/nu12092568

**Published:** 2020-08-25

**Authors:** Rafael Salto, María D Girón, Carolina Ortiz-Moral, Manuel Manzano, Jose D Vílchez, Francisco J Reche-Perez, Pilar Bueno-Vargas, Ricardo Rueda, Jose M Lopez-Pedrosa

**Affiliations:** 1 Department of Biochemistry and Molecular Biology II, School of Pharmacy, University of Granada, Campus de Cartuja, 18071 Granada, Spain; rsalto@ugr.es (R.S.); ecom@correo.ugr.es (C.O.-M.); e.damaso@go.ugr.es (J.D.V.); freche@correo.ugr.es (F.J.R.-P.); 2 Abbott Nutrition R&D, Abbott Laboratories, 18004 Granada, Spain; manuel.manzano@abbott.com (M.M.); pilar.bueno@abbott.com (P.B.-V.); ricardo.rueda@abbott.com (R.R.); jose.m.lopez@abbott.com (J.M.L.-P.)

**Keywords:** catch-up growth, catch-up fat phenotype, insulin-resistance, metabolic flexibility, slow digesting carbohydrates

## Abstract

A nutritional growth retardation study, which closely resembles the nutritional observations in children who consumed insufficient total energy to maintain normal growth, was conducted. In this study, a nutritional stress in weanling rats placed on restricted balanced diet for 4 weeks is produced, followed by a food recovery period of 4 weeks using two enriched diets that differ mainly in the slow (SDC) or fast (RDC) digestibility and complexity of their carbohydrates. After re-feeding with the RDC diet, animals showed the negative effects of an early caloric restriction: an increase in adiposity combined with poorer muscle performance, insulin resistance and, metabolic inflexibility. These effects were avoided by the SDC diet, as was evidenced by a lower adiposity associated with a decrease in fatty acid synthase expression in adipose tissue. The improved muscle performance of the SDC group was based on an increase in myocyte enhancer factor 2D (MEF2D) and creatine kinase as markers of muscle differentiation as well as better insulin sensitivity, enhanced glucose uptake, and increased metabolic flexibility. In the liver, the SDC diet promoted glycogen storage and decreased fatty acid synthesis. Therefore, the SDC diet prevents the catch-up fat phenotype through synergistic metabolic adaptations in adipose tissue, muscle, and liver. These coordinated adaptations lead to better muscle performance and a decrease in the fat/lean ratio in animals, which could prevent long-term negative metabolic alterations such as obesity, insulin resistance, dyslipidemia, and liver fat deposits later in life.

## 1. Introduction

In humans, during the early period of life, in addition to genetic factors, the rate of growth is determined by nutrition. Low birth weight/poor neonatal growth is often associated with food intake restrictions and undernutrition in early childhood and can persist into adulthood in the absence of an appropriate nutritional intervention [1]. Macro- (carbohydrates, proteins, and lipids) and micronutrients are needed as building blocks and as a source of energy for a proper linear growth in children [2], and there are different physiological and pathological situations during childhood development in which nutritional intervention could help to avoid metabolic dysfunctions related to the processes of body growth and development. Besides, nutrients can be key regulatory factors of the metabolism, and growth faltering in children can occur at any stage from birth to adolescence, due to deficiencies in single nutrients, multiple micronutrients, macronutrients, energy, or more commonly, a combination of many nutritional deficiencies [3].

The term catch-up growth, defines a physiological adaptation that restores the genetically programmed growth upon refeeding. Catch-up growth, therefore, constitutes an effort to recover from the deleterious effects of poor growth on development and health. However, during the past few years, several large epidemiological studies have suggested that catch-up growth may also be a long-term health hazard [1]. An inadequate diet during the refeeding period has been correlated with a preferential catch-up growth, where an excessive fat deposition takes associated with hyperinsulinemia and impaired glucose homeostasis place (raising the so-called catch-up fat phenotype) [4]. Besides, an increased fat infiltration in the muscle raises concerns about long-term metabolic risks [1].

Insulin resistance is present during the fast rate of growth that takes place in the initial period of the catch-up growth. This idea is supported by a higher plasma insulin response to a glucose load found during catch-up growth in children born small-for-gestational-age and in children showing early adiposity rebound. Therefore, a cornerstone of the preferential catch-up fat is insulin resistance that is associated with metabolic inflexibility and suppressed thermogenesis [1,4]. It is essential to consider how insulin resistance affects the physiology, function, and regulation of the growth process in its main target tissues (bone, muscle, and adipose) to prevent this thrifty “catch-up fat” phenotype and to search for ingredients that restore the benefits associated with healthy catch-up growth [5].

Dietary carbohydrates (CHO), one of the main sources of energy in infancy and childhood, are essential for growth and development. In addition to the amount of CHO, it is important the quality of the CHO consumed, measured by its glycemic index (GI). GI classifies carbohydrate-containing foods according to the postprandial glucose response. A higher index correlates with a faster postprandial serum glucose increase, and a rapid insulin response. A fast response to insulin leads to rapid hypoglycemia, which is associated with hunger and a higher caloric intake. Conversely, a low GI diet corresponds to a slower absorption of CHO and consequently with lower blood glucose fluctuations, as a marker of better glycemic control [5,6].

This work has aimed to evaluate the effect of diets with different carbohydrate composition and digestibility in animals that had experienced nutritional growth retardation after weaning. In the re-feeding period, animals were fed diets that differ both in CHO content and type related to their GI. The effects of these diets were analyzed in three organs or tissues: adipose tissue, muscle, and liver. Fuel utilization, metabolic adaptation, and signaling pathways were measured in these tissues.

We have used a model of nutritional growth retardation, which closely resembles the incomplete nutrition observed in children who consume insufficient total energy to sustain normal growth and weight gain. In this model, the refeeding with a diet containing slowly digestible CHO (SDC) prevents the harmful effects of a preferential catch-up fat phenotype after an early protein-caloric restriction.

In this experimental setting, the SDC diet produces a decrease in adiposity and an increase in muscle performance compared with the use of a rapid digestible CHO diet (RDC). These effects are associated with an increase in fatty acid oxidation relative to carbohydrate oxidation, parallel to a reduction in resistance to the action of insulin. In adipose tissue, the SDC diet promotes a decrease in lipid synthesis. In the muscle, the group that received this diet showed a higher expression of the glucose transporter 4 (GLUT4), a better oxidative use of glucose associated with greater muscle differentiation and better performance. Finally, in the liver, the SDC diet promoted glycogen storage and decreased fatty acid synthesis. Taken together, the animals that were fed this SDC diet showed a better insulin sensitivity, muscular performance, and lower adiposity than the animals fed the RDC diet.

## 2. Materials and Methods

### 2.1. Housing

Thirty weanling male Sprague Dawley rats (Oncin France Strain A (OFA); 21–25 days old) were provided by Charles Rivers (Orleans Cedex, France). Animals were individually housed in cages and kept under 12 h light-12 h dark cycles. The room temperature was maintained at 21 °C. All experimental procedures (approval code 23/05/2016/088) were carried out according to the ‘European Convention for the Protection of Vertebrate Animals used for Experimental and other Scientific Purposes’ (Council of Europe No 123, Strasbourg 1985), as well as to the ethical guidelines for animal experimentation provided by the Spanish National Research Council (RD 1201/2005 10 October).

### 2.2. Experimental Design

**Scheme 1:**
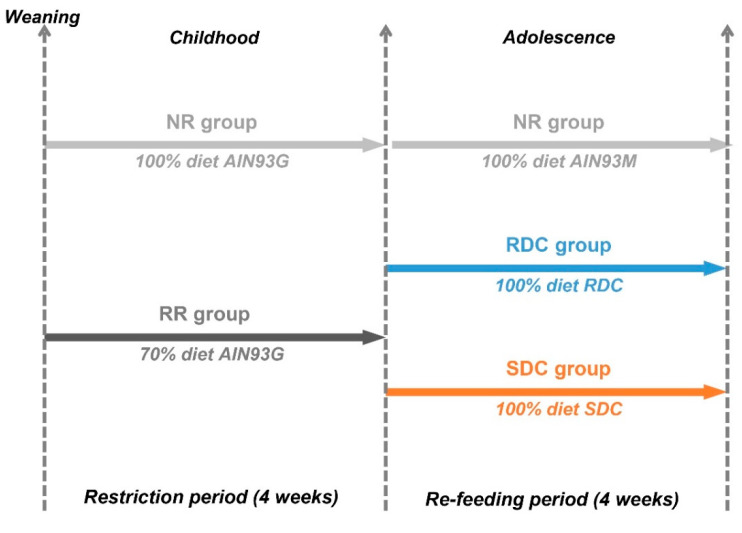
Schematic drawing of the experimental design used in this study. NR group, not restricted; RDC group, re-feeding with rapid digestible carbohydrates (CHO) diet; SDC group, re-feeding with slow digestible CHO diet.

Scheme 1 shows the experimental design. A total of thirty rats were randomly assigned to two groups: a non-restricted rat group (NR, *n* = 10) was fed with a rodent standard diet (AIN93G: 7.0% fat, 17.8% protein, 67.4% carbohydrates and 4.8% fiber per weight) [7] ad libitum, while a restricted rats group (RR, *n* = 20) received 70% of the amount of food consumed by NR the previous day, corrected by body weight (food intake in g/100g body weight per day).

After 4 weeks of food restriction, RR rats were divided into two groups of ten animals each and were fed ad libitum for 4 weeks with different humanized experimental diets regarding their carbohydrate composition (Table 1). The NR group was fed ad libitum a standardized rodent diet (AIN93M). All rats had free access to water. Bodyweight (BW) and dietary intake were recorded every day before food distribution. Body length (BL) and composition were registered every 4 weeks.

Ten rats of each group were sacrificed at the end of the re-feeding period in post-absorptive conditions (1 h after oral meal tolerance challenge with the experimental diets; 10 kcal diet/kg BW). Blood samples were isolated before and after oral gavage. Blood was collected either into serum tubes or in tubes containing anticoagulant EDTA (for isolating plasma). Animals were sacrificed by exsanguination under intramuscular anesthesia. Tissues were immediately isolated, weighed, and snap-frozen in liquid nitrogen and kept at −80 °C for posterior analysis.

### 2.3. Body Composition

Body composition, body fat mass, and soft lean body mass were evaluated by quantitative nuclear magnetic resonance imaging (EchoMRI 700 system; Echo Medical Systems, Houston, TX, USA). EchoMRI technique has been established and refined to minimize discomfort to the animals without the need to anesthetize the animal.

### 2.4. Indirect Calorimetry

At the end of the study, rats were introduced in a Phenomaster Indirect Calorimetry System (TSE System, Bad Homburg, Germany) for 24 h. Rates of oxygen consumption (VO2) and carbon dioxide production (VCO2) were calculated by TSE software and used to calculate respiratory quotation (RQ).

RQ, defined as the ratio between VCO2 and VO2, was used to determine the relative participation of glucose and lipids in energy production. A ratio close to 1 indicates increased carbohydrate oxidation over fat oxidation, whereas a lower ratio (close to 0.7) reflects an increased fatty acid oxidation relative to carbohydrate oxidation [8,9]. Based on a modified Lusk table [10], the approximate use of each energy source by the different groups was calculated.

### 2.5. Biochemical Parameters

Serum/plasma was collected and parameters such as glucose, triglycerides, were analyzed using a clinical chemistry analyzer Pentra 400 (Horiba ABX, Montpellier, France). Insulin concentration in plasma was measured by Bioplex 200 system using the Milliplex Map Rat Metabolic Magnetic Bead Panel kit (Millipore; Billerica, MA, USA). Serum interleukin 6 (IL-6) and tumor necrosis factor α (TNF-α) were also assayed using the Bioplex 200 system. The assays were performed in a 96-well plate according to product instructions.

### 2.6. Western Blot Analysis

Tissue extracts were obtained using the following lysis buffer: 50 mM Hepes pH 7.5, 150 mM NaCl, 1% Nonidet P-40, 10 mM NaF, 20 mM NaPPi, 1 mM MgCl_2_, 1 mM CaCl_2_, 20 mM β-glycerophosphate, 2 mM sodium orthovanadate, 2 mM EDTA, 2 mM PMSF, 4 μg/L leupeptin tissues were homogenized for 15 s in Polytron at setting #4. The homogenate was centrifuged at 16,000× *g* for 15 min at 4 °C. The supernatant was transferred to a new Eppendorf tube (1.5 mL) and sonicated for 15 s (cycle 0.5, amplitude 70%). Samples from rat gastrocnemius muscle were first pulverized using a mortar and liquid nitrogen and then subjected to the above homogenization protocol. Protein concentration of the samples was measured using the bicinchoninic acid method [11]. Specific antibodies against GLUT4 (Biogenesis Ltd., Poole, UK); total and phospho (Ser473)-Protein Kinase B/Ak strain transforming (PKB/Akt) and Myocyte enhancer factor 2D (MEF2D) (Cell Signaling, Beverly, MA, USA); carbohydrate-responsive element-binding protein (ChREBP), fatty acid synthase (FAS), glucose transporter 1 and 2 (GLUT1 and GLUT2), sterol regulatory element-binding proteins (SREBP) SREBP1 and SREBP2, pyruvate kinase (PKM1/2), pyruvate dehydrogenase kinase (PDK4), ATPase5B, uncoupling proteins (UCP) UCP1 and UCP2 (Santa Cruz Biotechnology (Dallas, TX, USA) were used. Pyruvate carboxylase (PC) antibody was raised at Dr. Salto’s laboratory. Glyceraldehyde phosphate dehydrogenase (GAPDH) (Santa Cruz Biotechnology) was used as a load control. Data were normalized using the values of the reference animals as 100%.

### 2.7. Quantitative Real-Time PCR

Total RNA was isolated from adipose tissue, muscle, and liver using the TRIzol Reagent following the user guide instructions (Thermo Fisher Scientific, Madrid, Spain). The amount of total RNA was measured using a NanoVue Plus Spectrophotometer (GE Healthcare Life Sciences, Madrid, Spain) and the quality was verified by 1% agarose gel electrophoresis. Moreover, 1 μg of total RNA from each sample was reverse-transcribed using Maxima First Strand cDNA Synthesis Kit for RT-qPCR (Thermo Fisher Scientific, Madrid, Spain). qPCR was performed using specific primers on a MiniOpticon Real-Time PCR System (Bio-Rad, Madrid, Spain) with Bio-Rad CFX Manager 3.1 software and fluorescence signal detection (SYBR Green) after each amplification cycle. Sequences of the primers were for Carnitine palmitoyltransferase 1B (Cpt1b), forward 5′-TATTAAGAACACGAGCCAAC-3′ and reverse 5′-GTAGCAAGTCTGTCTCTTTG-3′; glyceraldehyde phosphate dehydrogenase (GAPDH), forward 5′-GACATGCCGCCTGGAGAAAC-3′ and reverse 5′-AGCCCAGGATGCCCTTTAGT-3′; pyruvate dehydrogenase kinase (Pdk4), forward 5′-CTATTCAAGAATGCCATGAGG-3′ and reverse 5′-GTATGTGTAACTAAAGAGGCG-3′; peroxisome proliferator-activated receptor delta (ppard), forward 5′- CATAACGCACCCTTCATC-3′ and reverse 5′-GATGTTCTTGGCGAACTC-3′; peroxisome proliferator-activated receptor gamma (pparg), forward 5′-AAGACAACAGACAAATCACC-3′ and reverse 5′- CAGGGATATTTTTGGCATACTC-3′. First strand cDNA was used as a template in 10 μL reactions including 5 μL of 2x iTaq Universal SYBR Green supermix and 500 nM of each primer. Negative controls (with no DNA template) for each primer set were included in each run. qPCR cycling was performed at 95 °C (2 min), followed by 40 cycles at 95 °C (15 s), 57 °C (30 s), and, finally, a melt curve program (60–95 °C with a heating rate of 0.1 °C/s and a continuous fluorescence measurement). Measurements of gene expression concerning each RNA extraction were obtained in triplicate. Relative expression of mRNA was calculated using the 2−ΔCt method with glyceraldehyde-3-phosphate dehydrogenase (GAPDH) as an internal reference gene.

### 2.8. Glycogen Quantification

Hepatic and muscle glycogen was isolated as described [12]. Tissue homogenates (10%) were made in 0.03 N HCl and spread evenly on pieces of filter paper (Whatman 3 MM chromatography paper, 2.0 × 2.0 cm) in duplicate. The papers were dropped immediately into a beaker containing 66% EtOH stirred gently by a rotating magnet screened from the papers by a wire mesh. The papers were subsequently washed three times for 40 min in 66% EtOH. They were then briefly rinsed with acetone and dried under a stream of warm air. The dried filter papers were cut into four pieces and placed in a tube containing 0.4 mL of 0.2 M acetate buffer, pH 4.8; 0.2 mg of amylo-α-1,4-a-1,6-glucosidase; and H2O to a final volume of 2 mL. The vials were incubated for 90 min at 37 °C with gentle shaking. Appropriate controls were prepared by incubating aliquots of homogenate in acetate buffer minus amyloglucosidase. Glucose content in the incubated samples was determined by the glucose oxidase method.

### 2.9. Quantitative Determination of Creatine Kinase (CK)

CK activity was determined using a CK-NAC kit (Spinreact). CK catalyzes the reversible transfer of a phosphate group from phosphocreatine to ADP. This reaction is coupled to those catalyzed by hexokinase and glucose-6-phosphate. The rate of NADPH formation, measured spectrophotometrically, is proportional to the catalytic concentration of CK present in the sample.

### 2.10. Statistical Analysis

Results were expressed as means ± SEM. The statistical significance of variations was evaluated using one- or two-way ANOVA, or the non-parametric test depending on the homoscedasticity test (Bartlett’s test). Post hoc paired comparison, using Tukey’s test or Dunn’s test, was performed to check for significantly different effects between all pairs of diets. A *p*-value < 0.05 was considered significant.

## 3. Results

This work has aimed to evaluate the effects of a dietary intervention in an animal model of nutritional growth retardation and catch-up growth. In this model, three experimental groups were defined (Scheme 1). A group termed Non-Restricted (NR) in which the animals were fed at libitum during both phases of the experiment (8 weeks). This reference group has been compared to other groups in which a protein-caloric restriction was implemented at weaning for 4 weeks and then were re-fed, for additional 4 weeks, either with a Rapid Digestible Carbohydrate diet (RDC group) or a Slow Digestible Complex Carbohydrate diet (SDC group).

### 3.1. Experimental Catch-up Grow Model

**Figure 1:**
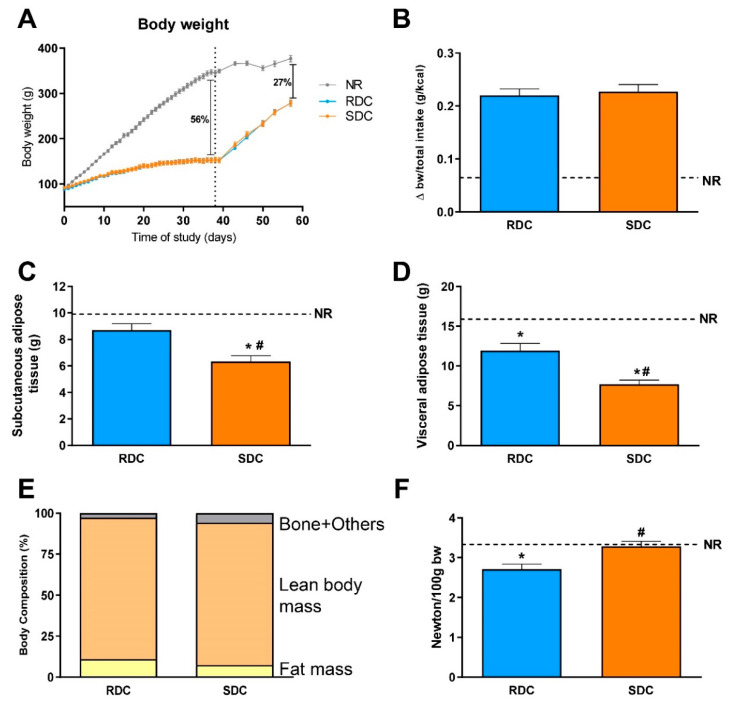
Catch-up growth in the experimental model. (**A**). Body weight evolution. (**B**). Food efficiency. Subcutaneous (**C**) and visceral (**D**) fat deposits. (**E**). Body composition of the RDC and SDC groups. (**F**). Grip strength. NR group, not restricted; RDC group, re-feeding with rapid digestible CHO diet; SDC group, re-feeding with slow digestible CHO diet. * Significant difference with NR group, *p* < 0.05. # Significant difference with RDC group, *p* < 0.05.

As expected, at the end of the food restriction period, the bodyweight of animals that received 70% of the diet consumed by NR showed a significant reduction in the body weight gain as compared to the healthy group (~56% reduction) (Figure 1A). During the re-feeding period, both dietary interventions induced a significant increase in body weight gain (+80% increment). During the re-feeding period, no significant differences in body weight gain and food efficiency [calculated as g bodyweight (BW) gain/Kcal consumed] were detected between both experimental dietary groups (Figure 1B).

More interesting, significant differences were obtained regarding the percentage of total body fat, subcutaneous, and visceral fat between the RDC and SDC groups (Figure 1C–E). The comparison between RDC y SDC groups showed a decrease of up to 33% in fat mass and an increase of 0.5% in lean body mass in the SDC group (Figure 1E).

Therefore, the results obtained from the RDC group indicated that the increase in body weight gain during the re-feeding period translated in an increase in fat tissue rather than in lean body mass when compared to the SDC group. Moreover, when the grip strength of the different experimental groups was assayed as a marker of muscle performance, the results indicated that there is again a better performance in the SDC group compared to the RDC one (Figure 1F).

**Figure 2:**
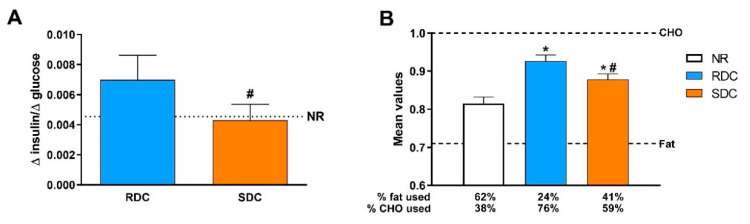
Insulin sensitivity and Energy substrate utilization in the experimental groups. (**A**). Insulin to glucose ratio. (**B**). Indirect calorimetry in the Catch-up growth experimental model. The basal respiratory quotient is shown. NR group, not restricted; RDC group, re-feeding with rapid digestible CHO diet; SDC group, re-feeding with slow digestible CHO diet. * Significant difference with NR group, *p* < 0.05. # Significant difference with RDC group, *p* < 0.05.

Next, since there is a fat mass increase on the RDC group, serum insulin and glucose measurements (Figure 2A) and calorimetry studies were conducted to determine the energy substrate utilization in the experimental groups (Figure 2B). Albeit fasting glucose and insulin levels and 1h postprandial glucose levels did not differ between RDC and SDC groups, for the RDC animals the insulin/glucose ratio (Figure 2A) was significantly higher compared with the SDC and NR groups.

Respiratory quotient (RQ), as the ratio between oxygen consumption levels and carbon dioxide production, was measured (Figure 2B) to reflect the ratio of carbohydrate to fat oxidation. A ratio close to 1 indicates an increase in carbohydrate oxidation over fat oxidation, while a lower ratio (close to 0.7) reflects an increase in fatty acid oxidation relative to carbohydrate oxidation [8]. We observed that the RDC group mainly (approx. 75%) obtained the energy through the oxidation of CHOs in contrast with the SDC group, which maintained a more balanced oxidation of CHO and fat to produce energy.

To explain the changes in fat deposition, muscle performance, insulin sensitivity, and RQ found between the RDC and SDC groups, metabolism, and signaling pathways were analyzed in the key tissues involved, adipose tissue, muscle, and liver.

### 3.2. Dietary Effects on Gene Expression, Signaling and Metabolic Pathways in Adipose Tissue

**Figure 3:**
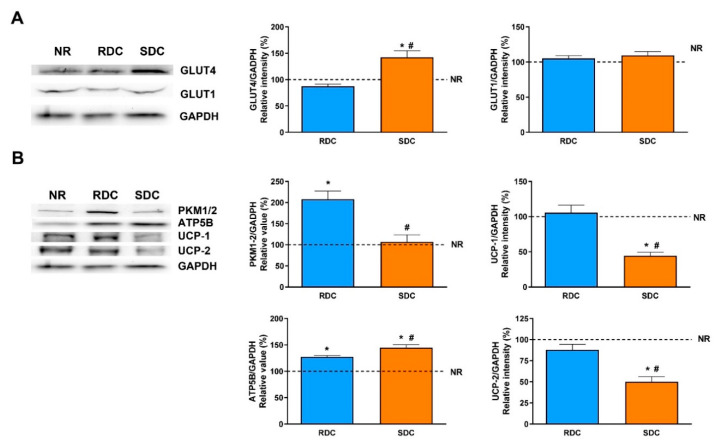
Adipose tissue expression of glucose transporters and enzymes in the experimental groups. (**A**). Glucose transporters. (**B**). Enzymes related to the glycolytic pathway. NR group, not restricted; RDC group, re-feeding with rapid digestible CHO diet; SDC group, re-feeding with slow digestible CHO diet. * Significant difference with NR group, *p* < 0.05. # Significant difference with RDC group, *p* < 0.05.

In adipose tissue, glucose transporters expression, GLUT1 and GLUT4, were analyzed by Western blot. Figure 3A shows that while there were no significant changes in the amount of the basal transporter GLUT1 among the different experimental groups, a significant increase in the expression of the insulin-dependent GLUT4 transporter was detected in the SDC group compared to the RDC and NR groups. Despite the higher levels of GLUT4 on the SDC group, the SDC rats showed significantly lower basal glucose oxidation compared to RCD rats measured as pyruvate kinase isoenzyme M (PKM1/2) expression (Figure 3B). These changes observed in the PKM1/2 were also associated with changes in the expression of ATP synthase and uncoupling proteins. While ATP synthase expression was significantly higher in the SDC group than in the RDC rats, the levels of mitochondrial uncoupling proteins were significantly lower in the SDC group compared to the RDC rats.

**Figure 4:**
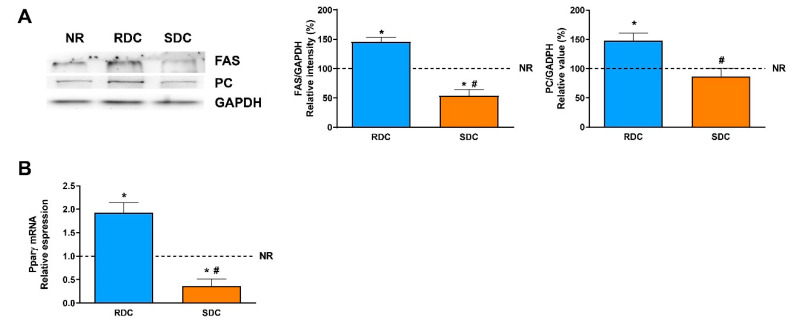
Lipogenesis in adipose tissue of the different experimental groups. (**A**) The expression of two lipogenic enzymes was assayed by western blot. (**B**) The expression of mRNA levels corresponding to Peroxisome proliferator-activated receptor (Pparγ) was assayed by qPCR as described in the methods section. NR group, not restricted; RDC group, re-feeding with rapid digestible CHO diet; SDC group, re-feeding with slow digestible CHO diet. * Significant difference with NR group, *p* < 0.05. # Significant difference with RDC group, *p* < 0.05.

Since an increase in adiposity has been detected in the RDC group compared with the SDC one, the expression of key enzymes related to lipogenesis was measured. Expression of fatty acid synthase (FAS) and pyruvate carboxylase (PC), as an anaplerotic enzyme related to the novo fatty acid synthesis, were analyzed (Figure 4A).

FAS expression in the SDC group was significantly lower compared to the NR and RDC groups. Moreover, FAS expression in the RDC group was significantly higher than in the control NR group. Furthermore, the profile of the expression of the PC, as the main anaplerotic enzyme in the channeling of glucose to TG, mimicked the FAS expression in the experimental groups.

In adipose tissue, adipogenesis is regulated by transcription factors such as peroxisomal proliferator-activated receptors (PPARs), being PPARγ especially relevant for the regulation of genes involved in fatty acid synthesis [13]. Our results (Figure 4B), indicated that while Pparγ mRNA was up-regulated in the RDC group compared with the control NR group, the SDC diet produced a significant decrease in its expression compared with the control NR and RDC groups.

### 3.3. Dietary Effects on Gene Expression, Signaling and Metabolic Pathways in Skeletal Muscle

**Figure 5:**
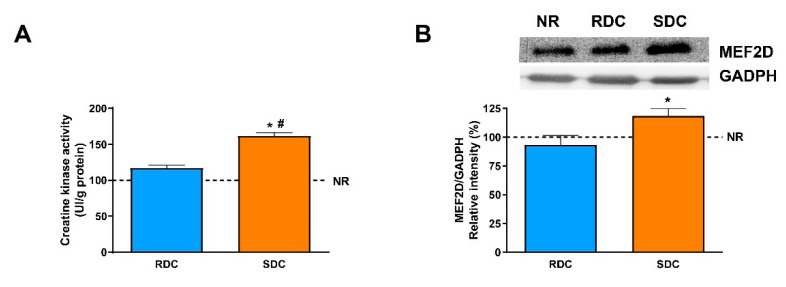
Markers of muscle performance. (**A**). Creatine kinase activity and (**B**) myocyte enhancer factor 2D (MEF2D) expression as markers of muscle performance and differentiation. NR group, not restricted; RDC group, re-feeding with rapid digestible CHO diet; SDC group, re-feeding with slow digestible CHO diet. * Significant difference with NR group, *p* < 0.05. # Significant difference with RDC group, *p* < 0.05.

Since an increase in grip strength was detected in the SDC group compared with the RDC one (Figure 1F), creatine kinase activity and the myocyte enhancer factor 2D (MEF2D) were measured as markers of muscle functionality. The higher levels of creatine kinase activity and MEF2D as a late marker of muscle differentiation (Figure 5) detected in the SDC compared with the RDC group support the idea of a better muscle performance.

**Figure 6:**
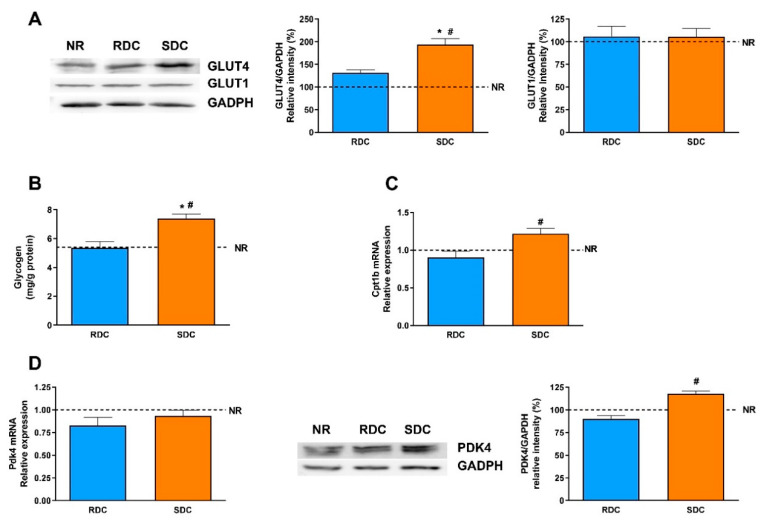
Muscle expression of key transporters and regulatory enzymes in the experimental groups. (**A**). Glucose transporters, (**B**) glycogen content, (**C**) Carnitine palmitoyl-transferase 1b (Cpt1b) mRNA expression. (**D**). Pyruvate dehydrogenase kinase 4 (PDK4) mRNA and protein expression. NR group, not restricted; RDC group, re-feeding with rapid digestible CHO diet; SDC group, re-feeding with slow digestible CHO diet. * Significant difference with NR group, *p* < 0.05. # Significant difference with RDC group, *p* < 0.05.

Next, key metabolic enzymes and transporters were assayed in skeletal muscle. First, the expression of GLUT1 and GLUT4 was measured (Figure 6A). In muscle, there were no significant changes in the expression of the basal transporter GLUT1 among the experimental groups. On the contrary, GLUT4 was overexpressed in the SDC group compared with the control NR and RDC groups. The increased glucose uptake in the SDC group was paralleled to an increase in muscle glycogen content in this group compared to the other experimental groups (Figure 6B). On the contrary, no significant differences among groups were observed when PKM1/2, ATPase5b subunit, or uncoupling proteins (UCP), such as UCP2 proteins, were assayed (Supplemental Material Figure S1).

In addition to a better muscle performance, our data from calorimetric studies indicated changes in the fuel utilization in the basal state among dietary groups (Figure 2B). Therefore, the expression of carnitine palmitoyl-transferase 1b (Cpt1b) and pyruvate dehydrogenase kinase isoenzyme 4 (PDK4) as proteins responsible for controlling fuel use in muscle were measured. Cpt1b mRNA levels were higher in the SDC group compared to the RDC one (Figure 6C). Albeit PDK4 mRNA levels were similar among groups, there was a significant increase in PDK4 when measured as protein in the SDC group compared with the RDC (Figure 6D).

**Figure 7:**
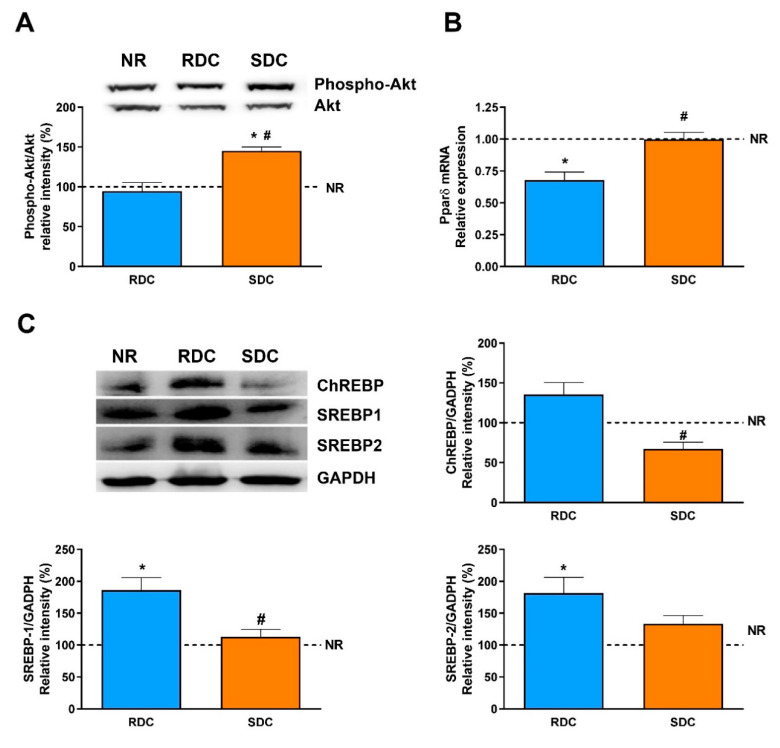
Signaling pathways and differentiation in muscles of the different experimental groups. (**A**). Protein Kinase B/Ak strain transforming (PKB/Akt) phosphorylative status and (**B**) peroxisome proliferator-activator receptor gamma (Pparδ) expression. (**C**). Expression of carbohydrate response element binding protein (ChREBP), steroid response element binding proteins (SREBP1 and SREBP2). NR group, not restricted; RDC group, re-feeding with rapid digestible CHO diet; SDC group, re-feeding with slow digestible CHO diet. * Significant difference with NR group, *p* < 0.05. # Significant difference with RDC group, *p* < 0.05.

In muscle, as key signaling elements the phosphorylative status of PKB/Akt as well as the mRNA levels of peroxisome proliferator-activator receptor delta (Pparδ) were measured (Figure 7). Animals from the SDC group showed significant increases in phosphorylated Akt/PKB kinase and Pparγ expression. In muscle, carbohydrate response element binding protein (ChREBP) levels have been associated with alterations in glycolytic/oxidative enzymes [14] and our results indicated a significant decrease in the SDC group. Furthermore, when the expression of steroid response element binding proteins (SREBP1/2), involved in lipid metabolism [15], is analyzed, a similar result was found.

### 3.4. Dietary Effects on Gene Expression, Signaling and Metabolic Pathways in Liver

**Figure 8:**
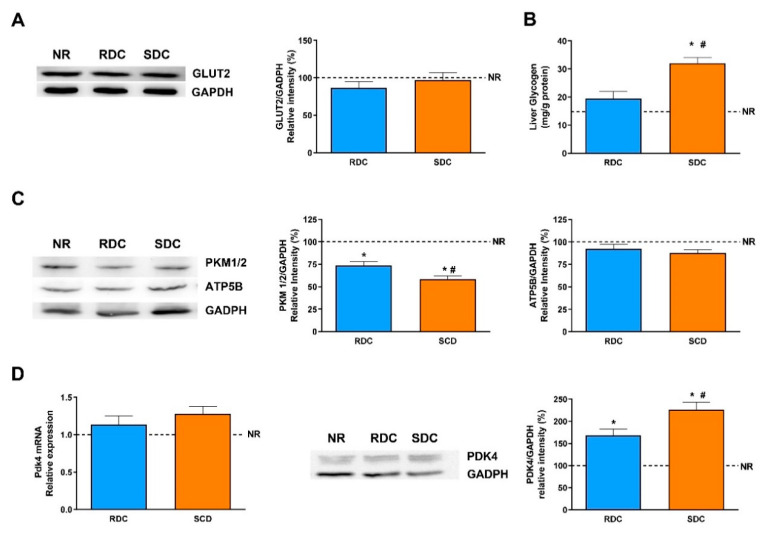
Liver glucose transporter, glycogen levels, and expression of key carbohydrate metabolism regulatory enzymes in the experimental groups. (**A**) Glucose transporter 2 (GLUT2), (**B**) glycogen content, (**C**) pyruvate kinase (PKM1/2) and (ATP synthase 5b subunit (ATP5b). (**D**) Pyruvate dehydrogenase kinase 4 (PDK4) mRNA and protein expression. NR group, not restricted; RDC group, refeeding with rapid digestible CHO diet; SDC group, refeeding with slow digestible CHO diet. * Significant difference with NR group, *p* < 0.05. # Significant difference with RDC group, *p* < 0.05.

Once analyzed some of the key metabolic elements in adipose tissue and muscle, the effects of the different diets on the liver were studied. First, GLUT2 mediated glucose uptake, liver glycogen deposits, and glycolytic enzymes were assayed (Figure 8).

**Figure 9:**
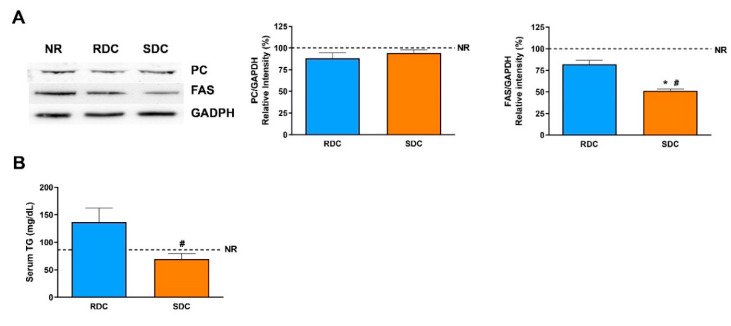
Lipogenesis in liver of the different experimental groups. (**A**) The expression of two lipogenic enzymes were assayed by western blot. (**B**) Circulating triacylglycerols (TG) in fasting were measured. NR group, not restricted; RDC group, refeeding with rapid digestible CHO diet; SDC group, refeeding with slow digestible CHO diet. * Significant difference with NR group, *p* < 0.05. # Significant difference with RDC group, *p* < 0.05.

Albeit, there were no significant changes among groups in the GLUT2 expression, animals fed the SDC diet showed a higher amount of glycogen, associated with decreased PKM1/2 activity and an increase in PDK4 amount compared with the RDC. These results pointed out to a change of the liver carbohydrate metabolism to glycogen synthesis rather than to the use of glucose as a lipogenic substrate. To confirm the decreased use of glucose in the liver to synthesize triglycerides, the amount of PC and FAS as the main lipogenic enzymes was assayed (Figure 9A). Our results indicated that while there were no significant changes in PC expression among groups, the expression of FAS is significantly lower in the SDC group compared to the other experimental groups. The changes of FAS levels in adipose tissue and liver were paralleled with the circulating TG levels in the fasted animals (Figure 9B).

**Figure 10:**
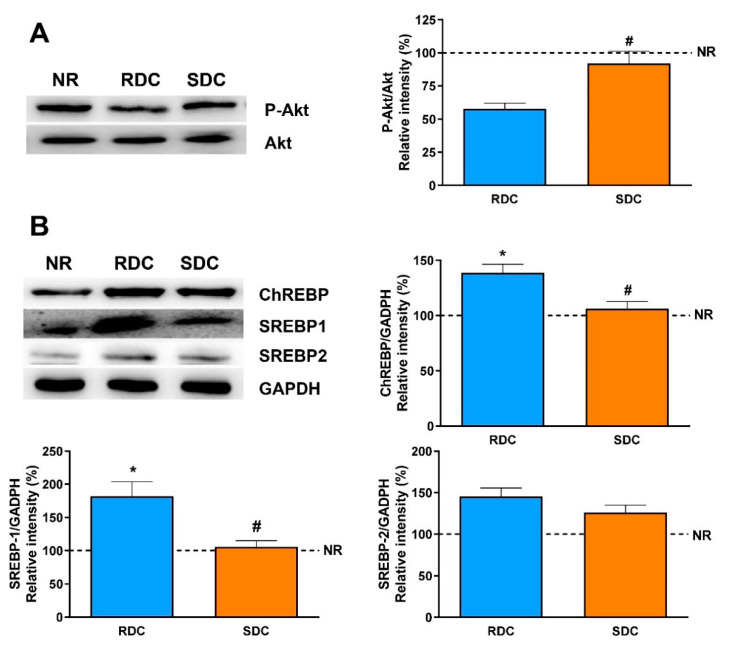
Signaling pathways in the liver of the different experimental groups. (**A**) Protein Kinase B/Ak strain transforming (PKB/Akt) phosphorylative status and (**B**) expression of carbohydrate response element binding protein (ChREBP), steroid response element binding proteins (SREBP1 and SREBP2). NR group, not restricted; RDC group, refeeding with rapid digestible CHO diet; SDC group, refeeding with slow digestible CHO diet. * Significant difference with NR group, *p* < 0.05. # Significant difference with RDC group, *p* < 0.05.

Key signaling elements were also measured in the liver. Figure 10A shows the phosphorylative status of Akt/PKB. Animals from the SDC group showed a significant increase in phosphorylated Akt/PKB kinase. Furthermore, ChREBP levels are especially relevant in the liver for the regulation of lipogenesis [14]. Our results indicate, in parallel with the muscle, a significant decrease in the transcriptional regulator levels in the SDC group. Furthermore, when the expression of SREBP1 protein, involved in association with ChREBP in lipid metabolism, is analyzed, similar results were found (Figure 10B).

**Figure 11:**
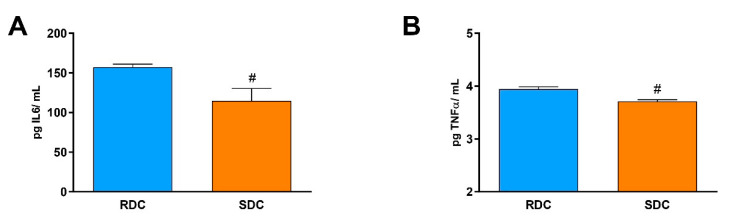
Inflammatory biomarkers in the RDC and SDC groups. Serum interleukin 6 (IL-6) (**A**) and tumor necrosis factor α (TNF-α) (**B**) levels have been determined for the RDC and SDC groups. RDC group, refeeding with rapid digestible CHO diet; SDC group, refeeding with slow digestible CHO diet. # Significant difference with RDC group, *p* < 0.05.

Finally, in addition to metabolic profile in rats under different diet protocols, inflammatory biomarkers were assayed in serum. Figure 11 shows IL-6 and TNF-α levels in the RDC and SDC groups. As expected, the RDC group showed higher cytokines levels compared with the SDC groups as indicative of a higher inflammatory status compared with the SDC group.

## 4. Discussion

Normal growth in humans is the consequence of interactions mainly between genetic, nutritional, and environmental mechanisms that lead to weight and height gain. However, different growth-retarding diseases and environmental conditions can promote a downward deviation from the normal growth curve leading to a hinder in the linear growth. When normal conditions take place again, exaggerated acceleration in linear growth characterized by increasing weight and height at a rate greater than the normal one for a given age can occur. This phase of catch-up growth” directs the children toward their pre-retardation growth curve [16]. Therefore, short-term catch-up growth has been considered beneficial in terms of body weight recovery [17].

However, the catch-up process is frequently associated with an excessive rate of body fat gain rather than muscle, termed “catch-up fat”. This phenotype has been associated with long-term negative consequences [18], as an increased risk of obesity, type 2 diabetes, and cardiovascular diseases. The mechanisms of the catch-up fat phenotype are unknown, but the adiposity could be due to a suppression of thermogenesis, skeletal muscle insulin resistance, and adipose tissue insulin hyper-responsiveness [19,20,21,22]. These changes that lead to a channeling of glucose utilization from skeletal muscle to de novo lipogenesis in adipose tissue, allows a rapid replenishment of fat storages.

In our experimental model, at weaning two groups of animals received 70% of the amount consumed by an unrestricted group for one month. Compared to unrestricted animals, restricted animal groups show an accelerated rate of weight gain upon refeeding, as a model of catch-up growth in undernourished children. Subsequently, restricted animals were fed for another month ad libitum with diets that differed in the composition of CHO, slow (SDC) or rapid (RDC) digestible CHO. The amount of soluble fiber provided by the SDC diet was higher than RDC. Several investigations in adults have identified the potential role of soluble/fermentable fiber in the control of body weight. These studies have suggested that the consumption of inulin-type fructans may induce a reduction in body weight by promoting satiety and decreasing spontaneous energy intake [23,24]. In obese children, similar effects have been described [25]. However, in undernourished children, a decrease in body weight is not advisable. In our study, although the SDC diet contains a higher amount of fiber, similar body weight gains were found with both experimental diets. The prevention of a decrease in body weight gain in the SDC group could be attributed to the use of a blend of soluble fibers, including resistant maltodextrin and inulin-type fructan that differ in their fermentation characteristics and therefore in their prebiotic effects. Therefore, our nutritional intervention has mainly enabled us to demonstrate the different effects of slowly vs. rapidly digestible CHO on catch-up growth and elucidate its underlying mechanism of action.

In agreement with the models of accelerated growth rate that related the catch-up fat phenotype with insulin resistance, our results point out that while the RDC diet caused insulin resistance measured as the blood insulin/glucose ratio (Figure 2A), the SDC diet normalized this parameter. Associated with the catch-up fat phenotype and driven by associated insulin resistance, changes in fuel use have been detected in pre-pubertal boys and girls with stunting. Stunted children show lower resting energy expenditure associated with a higher respiratory quotient for carbohydrate oxidation. On the contrary, in these children, fat oxidation to obtain energy is decreased [26]. Our results were similar since there is an increase in CHO catabolism in the RDC group that is normalized in the SDC animals measured as respiratory quotient (Figure 2B). Parallel to these changes in CHO utilization, the SDC group showed increased use of lipids as an energy source compared to the RDC group. Changes in fuel use are closely related to fat deposition in the experimental groups. We observed preferential subcutaneous and visceral fat deposition in undernourished animals that consumed RDC as compared to SDC.

Catch-up fat phenotype not only affects adipose tissue but also has a strong effect on muscle fuel use and therefore muscle performance. In this situation, fuels are channeled to adipose tissue instead of muscle. This is due to the onset of muscle insulin resistance that led to a decrease in muscle glucose uptake and performance. Our results indicate that the animals from the SDC group have a better muscle performance measured by grip strength assay (Figure 1E) when compared to the RDC group. Furthermore, the SDC animals showed a higher muscle differentiation measured by CK and MEF2D levels (Figure 5).

Up to this point, while the RDC group shows all the hallmarks described for a catch-up fat phenotype, this adverse situation is prevented in the SDC group that shows a decreased fat/lean body mass index, increased grip strength, and more balanced fuel usage. We hypothesize that in the SDC group, a trend to preferential use of fat instead of carbohydrates as metabolic fuel is established. To back these hypotheses, an analysis of metabolites, key regulatory enzymes, as well as signaling pathways in adipose tissue, muscle, and liver of the animals, was carried out.

Adipose tissue is characterized by the storage of triacylglycerols and, together with the muscle, by the removal of glucose after meals in a process mediated by the insulin-dependent transporter, GLUT4. GLUT4 expression is significantly higher in the SDC group than in the RDC and NR groups while the expression of GLUT1 is similar among groups. From this data, we can conclude that the protein-caloric restriction and re-feeding of the animals with the SDC diet has not produced peripheral insulin resistance compared to the RDC group. In humans and rodents, GLUT4 expression in adipose tissue is decreased in states of insulin resistance such as diabetes and obesity [27,28,29] or response to a prolonged fasting. A situation of insulin resistance and down-regulation of adipose GLUT4 in diabetes and fasting can lead to changes in adipocyte substrate flux, such as increased lipolysis with the release of fatty acids and glycerol that is used for hepatic gluconeogenesis [30].

In adipose tissue, glucose is mainly used for the novo synthesis of fatty acids. There are significant differences in the use of glucose between the two groups that were fed the carbohydrate- enriched diets. While the RCD rats showed a higher glycolytic use of the glucose (measured as the expression of PKM1/2), the SDC rats showed a significantly lower glycolytic flow. The reduced glycolytic flow in the SDC group is a first insight of the reduced capacity of transformation of glucose into fatty acids and triglycerides in this experimental group. Furthermore, key lipogenic enzymes expression, PC, and FAS, was analyzed. PC is essential for several pathways, including lipogenesis. In adipose tissue, the role of PC in lipogenesis is to provide acetyl groups required for the novo fatty acid synthesis [13]. Our results show that catch-up growth produced a higher expression of the PC in the RDC group and that this negative effect was normalized to control NR group values in the SDC group.

Fatty acid synthase is the key regulatory enzyme in the synthesis of fatty acids. The expression of FAS in the three groups of rats is parallel to the PC expression. FAS levels are significantly lower in rats fed the SDC diet. Therefore, while catch-up growth using the RCD diet leads to an increase in adipogenesis due to a higher expression of PC, providing acetyl-CoA units, plus an increase in FAS levels, none of these undesirable effects are detected in the SDC group.

In the SDC group, there was a lower glycolytic flow coordinated with a minor ability to synthesize fatty acids. On the contrary, in this group, ATP synthase expression is significantly higher compared to the RDC group. Moreover, in the SDC group levels of uncoupling proteins are decreased compared to the other experimental groups. Uncoupling proteins (UCP1 and UCP2) catalyze the leak of protons across the mitochondrial membrane, which uncouples oxidative respiration from ATP synthesis, thus the energy derived from substrate oxidation is dissipated as heat [31]. UCP1 was primarily discovered in brown adipose tissue and it is best known for its role in adaptive non-shivering thermogenesis and control of body weight. Low temperature or over-feeding has been described to lead to the up-regulation of Ucp1 mRNA expression. Furthermore, UCP2 down-regulation also improves insulin resistance in white adipose tissue [32]. Both uncoupling proteins are significantly less expressed in the SDC group providing a more functional mitochondrial pathway for the aerobic oxidation of substrates. Taken together, an enhanced ATP synthase expression and a decreased uncoupling protein expression in the SDC group could indicate a more efficient use of glucose to obtain energy in the form of ATP to compensate for the reduced glycolytic flow.

In adipose tissue, insulin sensitivity and adipogenesis are regulated by transcription factors such as peroxisomal proliferator-activated receptors (PPARs) whose expression is also modulated by changes in glucose homeostasis and lipids [33]. PPARγ regulates the expression of genes required for endogenous cholesterol, fatty acid, and triacylglycerol synthesis; besides, PPARγ is considered essential to adipocyte differentiation. Our results indicate that catch-up growth in the RDC diet induced a significant increase in the expression of PPARγ that enables adipose lipid deposition. On the contrary, the SDC diet normalizes this signaling and in agreement with the key enzymes measured, normalizes fat deposition in the adipose tissue of the treated animals.

Taken together, in adipose tissue we found that the SDC diet increased GLUT4 expression that reflects better insulin sensitivity, but that is not associated with a higher adiposity. This is supported by data indicating that glycolytic flux is reduced (through a decrease in the PKM levels) as well as lipogenesis ability is also blocked (measured as a decrease in PC and FAS expression). These effects on the use of fuels by adipose tissue in the SDC group are probably mainly modulated by the PPARγ mediated pathway.

The use of SDC and RDC diets in restricted animals has also produced changes in muscle performance and insulin sensitivity. Skeletal muscle represents up to approximately 40% of the total body mass. Its main function is contraction-related, which is why it uses glucose as the main source of energy. The uptake of extracellular glucose in skeletal muscle increases under insulin-stimulation and exercise. In healthy subjects, skeletal muscle accounts for up to ∼80% of glucose disposal under insulin-stimulated conditions, as it occurs in the postprandial state; thus, revealing a basic role in glycemic homeostasis [34,35,36].

During the catch-up process, muscle metabolism and thermogenesis are reduced due to a diminished basal and insulin-stimulated PI3K activity [19]. This leads to a double effect, a decrease in muscle functionality, and a channeling of nutrients to the adipose tissue to increase adiposity [1,19]. Since muscle glucose uptake is mediated by the insulin-dependent GLUT4 and basal GLUT1 transporters, their total amounts were first assayed. Expression of GLUT4 is significantly higher in the SDC group and it indicates a higher glucose uptake to meet the energy needs of the muscle. GLUT4 is regulated by insulin that promotes an increase in its translocation to the plasma membrane. Since the SDC has lower circulating insulin levels, this result points out that the SDC diet may promote an improved insulin response in peripheral tissues. A higher glucose uptake in muscle has beneficial effects, it normalizes glycemia (that is also modulated in the SDC group by the increased liver glycogen storage) and provided highly metabolizable fuel to facilitate muscle contraction. As expected, the expression of the glucose basal transporter, GLUT1, does not change in response to nutritional conditions.

In muscle, glucose is intended to be stored as glycogen, or used to produce energy and mechanical strength. However, since the muscle lacks the enzyme glucose-6 phosphatase, muscle glycogen does not have the ability to regulate glycemia, and is only used as an internal energy storage. Our results suggest that feeding the SDC diet in caloric restricted animals tends to promote higher glycogen stores in the muscle compared with RDC and NR animals.

Reportedly, the skeletal muscle exhibits a noteworthy metabolic flexibility in the use of fuels in response to various challenges such as energy deprivation and changes in diet composition. Thus, in lean healthy people, under insulin stimulation, skeletal muscle can switch from fatty acid uptake and lipid oxidation to the suppression of lipid catabolism associated with an elevated glucose uptake that is oxidized or stored. On the contrary, obese and diabetic patients show greater rates of lipid oxidation in skeletal muscle that is relatively insulin-resistant [37]. More interesting, during exercise, muscle uses energy in the form of ATP to perform mechanical work and the energy is mainly obtained from the catabolism of glucose. In muscle, the preferred glucose catabolism is aerobic involving a mitochondrial net ATP synthesis. For this, an efficient respiratory chain coordinated with the ATP synthase is needed. Another relevant muscle feature is that in resting and during endurance exercise, a shift in substrate utilization from glucose toward fat occurs.

Our results indicate a higher expression of the ATP synthase in the SDC group compared to the other experimental groups. Moreover, the expression of mitochondrial UCP2 is similar in the three groups of animals. These data could indicate that there is no dissociation between oxidation and phosphorylation in the SDC group, and that the glucose stored as glycogen in the muscle is efficiently used to obtain energy for muscle performance. It is also interesting to point out that reduced gene expression of UCP2 in skeletal muscle has been observed in human obese subjects [38]. The increase of aerobic glucose metabolism in the muscle of the SDC group could lead to better muscle performance. In fact, for this experimental group, an increase in grip strength has been reported. As a confirmation of this observation, creatine kinase (CK) activity was assayed and a significant increase in the SDC group compared with the RDC and NR ones is detected. The increase in CK is probably related to an increase in muscle cell differentiation since MEF2D, a marker for muscle differentiation [39], showed higher levels in the SDC than in the other groups.

As previously stated, during endurance exercise a shift in substrate utilization occurs. In fact, in the SDC group, the calorimetry results support this metabolic adaptation. This process is highly dependent on muscle PPARδ, a nuclear receptor that serves as a key regulator of FA metabolism in muscle [40]. Therefore, we consider in our experimental setting two signaling pathways to regulate fuel use. One mediated by insulin and downstream kinases and a second one regulated by PPARγ expression. First, the phosphorylation status of Akt, a key kinase in this signaling pathway, is significantly higher in the SDC group. This result correlates with the enhanced muscle insulin sensitivity and expression of GLUT4. As it has been described [19] that a decreased PI3K signaling in skeletal muscle could be one of the main causes of resistance to the action of insulin during catch-up growth, our results indicate that the SDC diet could prevent these negative effects.

Overexpression of PPARδ can increase FA oxidation in skeletal muscle through the induction of two mitochondrial gatekeeper proteins, carnitine palmitoyl-transferase 1b (Cpt1b), the rate-limiting enzyme in the transport of FAs into the mitochondria and pyruvate dehydrogenase kinase isozyme 4 (Pdk4), which negatively regulates the influx of glucose-derived pyruvate into the mitochondrial TCA cycle [41]. The increase in the expression of PPARδ has also been involved in the down-regulation of glycolysis and transport of pyruvate to the mitochondria [40,42]. Furthermore, PPARδ signaling in muscle is associated with increased insulin sensitivity.

Therefore, we have determined the expression of ppard, Pdk4, and Cpt1b. The expression of PPARδ is significantly lower in the RDC group compared to the other two groups. There is also a concomitant increase in the expression of Pdk4 in the SDC group. The over-expression of Pdk4 indicates that there is a switch in the muscle of these animals toward the use of fatty acids as a metabolic fuel [35]. These results are also supported by the Cpt1b expression levels. Taken together, the increased values of PPARδ, Akt phosphorylation and Pdk4 are indicative of increased insulin sensitivity in muscle and an increase in the metabolic flexibility of the tissue.

In addition, it is well established that ChREBP (and its muscle isoform MondoA) is involved in the enhancement of muscle lipogenesis and muscle lipid stores that also lead to insulin resistance in the tissue [43]. In fact, in muscle from diabetic patients, ChREBP can be associated with an increase of glycolytic/oxidative enzymes [44]. Our results are in agreement with these antecedents, while an RDC diet promotes an increase in the ChREBP levels, the SDC can significantly reduce the amount of the transcriptional regulator, therefore reducing the risk on this experimental group of insulin resistance and metabolic inflexibility. Most of the genes regulated by ChREBP also show binding sites in their promoters for the SREBP family of transcriptional regulators in such a way that they act in a coordinated mode.

In muscle, activation of the Akt pathway induces the expression of SREBP-1, which would indicate other functions of these transcription factors in a tissue with low lipid synthesis. SREBP-1 overexpression is associated with a decrease in the expression of myogenesis regulatory factors, such as MEF2, which participates in muscle differentiation [45]. The higher activation of Akt and increased expression of MEF2D in the SDC group compared to the RDC one, support again these hypotheses and shown a beneficial effect of the SDC diet on muscle functionality.

The liver is responsible for the homeostasis of glucose and other metabolic fuels in the organism to provide energy and building blocks to other tissues. The body regulates glycemia harmonizing glucose production and storage in the liver and kidney, and by regulating its uptake in peripheral tissues as muscle and adipose tissue [35]. Moreover, the liver maintains metabolism homeostasis by processing dietary fat, carbohydrates, and proteins. It metabolizes glucose either to generate energy in the form of ATP or to obtain metabolic intermediates that will be substrates for the synthesis of fuels such as glycogen or TG [46,47].

In liver, glycogen content from the SDC group is higher than in the other two experimental groups, pointing out to a relevant role of the liver in the control of glycemia in response to this complex carbohydrate diet. Another positive effect of the SDC diet is that FAS in the liver shows a significant decrease compared to NR and RDC groups. FAS catalyzes the de novo synthesis of fatty acids. In the liver, FAS produces fat for storage of energy when nutrients are present in excess. Then, fatty acids are packed into Very Low Density Lipoproteins (VLDL) particles and transported to adipose tissue and other extrahepatic tissues [14]. The significant decrease of FAS in the liver in the SDC group could be considered as a protective effect toward future fat stores in the liver of the animals that have experienced the catch-up process. Circulating TG levels are significantly lower in the SDC group compared with the RDC group.

In addition, the measurement of PKM1/2 in the liver is relevant to determine the glucose metabolic fate since glucose (through the carbohydrate response element, ChORE) increases the transcription of different genes coding for glycolytic and lipogenic enzymes including PKM1/2 or FAS [48]. The expression of PKM is lower in the SDC group than in the RCD one. This result in the SDC group points out to an adaptive liver response that results in a decrease in the glycolytic flux and therefore, a lower use of glucose as metabolic fuel for TG precursors that is in concordance with the minor expression of FAS in this group.

Thus, it would seem that the carbohydrate composition of SDC mediates a metabolic state in which the decrease of blood insulin is combined with a higher storage of glucose as glycogen and at the same time a lower use of glucose by glycolysis and in consequence a decrease in the synthesis of triglycerides.

Metabolic flexibility is the capability of a system to regulate fuel (primarily glucose and fatty acids) oxidation in response to nutrient availability. Thus, the possibility to change substrate catabolism depending on the nutritional state depends on the steadiness between oxidation and storage capacities. Pyruvate dehydrogenase complex (PDC) plays a key role in the competition between fatty acids and glucose for oxidation. This complex is usually active in tissues in the fed state, but the suppression of its activity by pyruvate dehydrogenase (PDC) kinase (PDK) is critical to maintain energy homeostasis under nutritional conditions in which glucose is scarce and its synthesis is needed. PDK isoenzymes phosphorylate specific serine residues in the PDC [33]. Of all the known isozymes, PDK2 and PDK4 are the most widely distributed, and are highly expressed in the heart, liver, and kidney in humans and rodents. PDK4 is also abundant in pancreatic islets and in skeletal muscles that have high glucose utilization and fatty acid oxidation rates [35,49].

PDK4 is an enzyme that also controls glycolytic flux since the inactivation of PDC by PDKs can inhibit the conversion of pyruvate to acetyl-CoA, resulting in a shift of pyruvate to the TCA cycle or fatty acid synthesis toward gluconeogenesis. The expression of PDK4, as the widely distributed main isoform of PDK, has been measured at the level of transcription and translation. The protein levels of PDK4 are significantly higher in the SDC group compared with the other two groups. These results are again in concordance with the lower expression of PKM1/2 (glycolytic flux) and FAS (fatty acid synthesis).

The molecular bases of the liver metabolism regulation relies on a hepatic enhanced insulin sensitivity, since phospho-Akt levels are significantly higher in the SDC group compared with the RDC one. Furthermore, other concomitant signaling pathways such as the one regulated by ChREBP and SREBPs are also modified by the SDC diet to prevent a lipogenic programming of the metabolism.

Therefore, in the liver, we can conclude that, the effect of a diet with complex, slow digesting, and low glycemic index results in increased liver storage of glycogen and a decrease in hepatic FA synthesis. The expression of enzymes related to glycolysis and fatty acid synthesis as well as the content of PDK4 are in agreement with the above statement. These results point out to a change of the liver carbohydrate metabolism to glycogen synthesis rather than to the use as a lipogenic substrate preventing a liver storage of TG and decreasing blood TG.

Furthermore, the metabolic profile of the RDC group in adipose tissue, muscle, and liver is compatible with a pro-inflammatory status [50,51]. To confirm the negative effects of RDC diet on inflammation, as well as the preventive effects of the SDC diet, serum IL-6, and TNF-α levels, were assayed. The results (Figure 11) confirm the hypothesis that the nutritional approach proposed is able to minimize the inflammatory status in the experimental catch-up growth model assayed.

## 5. Conclusions

The effects of catch-up fat are remarkable and frequently derived in obesity, resistance to insulin action, and cardiovascular diseases later on. In this work, we have focused on the comparison of the dietary intervention in preventing the catch-up fat phenotype and promoting a healthy catch-up grow. Two diets were used that differ mainly in the slow (SDC) or fast (RDC) digestibility of the carbohydrates and their complexity. The results indicate that the SDC diet is better to prevent the catch-up negative effects of an early caloric restriction, reducing the deposits, and improving muscle functionality. The positive effects are present in all the organs and tissues analyzed. The metabolic effects observed in these organs work in a coordinated way to produce a more efficient fuel utilization and an increase in insulin sensitivity, in peripheral glucose uptake, and the channeling of glucose to an oxidative pathway rather than a lipogenic one. Overall, the SDC diet during catch-up growth may decrease obesity, metabolic syndrome, and diabetes later on by improving the metabolic plasticity in adipose, muscle, and liver.

## Figures and Tables

**Table 1:** Composition of diets used in this study.

Macronutrients	AIN93M	RDC	SDC
	NR	RDC	SDC
CHO (g/100 g diet)	78.5	67.0	67.0
Sucrose (g/100 g CHO)	13.0	32.0	
Isomaltulose (g/100 g CHO)			26.4
Sucromalt^®^ (g/100 g CHO)			22.1
Cornstarch (g/100 g CHO)	61.0		
MDs (g/100 g CHO)	20.0	64.0	23.0
IMOs (g/100 g CHO)			11.5
Resistant MD (g/100 g CHO)			10.0
Soluble fiber (g/100 g CHO)		4.0	7.0
Insoluble fiber (g/100 g CHO)	5.0		
Glycemic load (index)	1269	851	468
Protein (g/100 g diet)	14.0	15.0	15.0
Fat (g/100 g diet)	4.0	9.8	9.8

Glycemic load (GL) was calculated by first multiplying the amount of each carbohydrate contained in a daily dietary intake by its glycemic index (with the use of glucose as the reference food), then by summing the values from all carbohydrate (CHO) sources. Sucromalt^®^ is a slow digestive combination of sucrose and maltose. Daily dietary glycemic load thus represents the quality and quantity of carbohydrate intake and the interaction between the two. NR group, not restricted; RDC group, re-feeding with rapid digestible CHO diet; SDC group, re-feeding with slow digestible CHO diet. MD, maltodextrins; IMOs, isomalto-oligosaccharides.

## Supplementary Materials

The following are available online at https://www.mdpi.com/2072-6643/12/9/2568/s1, Figure S1: Muscle expression of enzymes related with the glycolytic pathway and mitochondrial ATP synthesis.
