# Supplementary material for: Dietary Complex and Slow Digestive Carbohydrates Prevent Fat Deposits During Catch-Up Growth in Rats
Source: Nutrients. 2020 Aug 25;12(9):2568. doi: 10.3390/nu12092568 (PMC7551611; doi:10.3390/nu12092568)
Supplement: Supplementary file 1 [file nutrients-12-02568-s001.pdf]

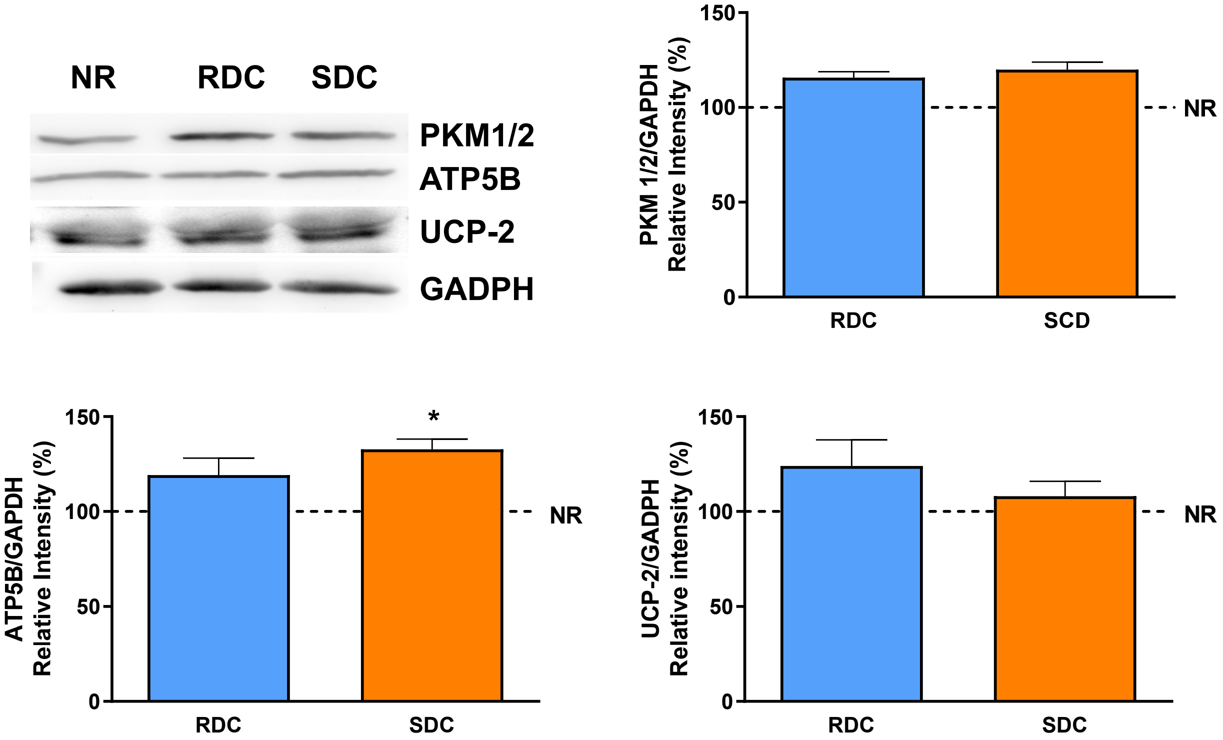

Supplemental Material Figure 1. *Muscle expression of enzymes related with the glycolytic pathway and mitochondrial ATP synthesis*. NR group, no restricted; RDC group, refeeding with rapid digestible CHO diet; SDC group, refeeding with slow digestible CHO diet. No Significant differences among groups were detected.
